# An innovative protocol to select the best growth phase for astaxanthin biosynthesis in *H. pluvialis*.

**DOI:** 10.1016/j.btre.2021.e00655

**Published:** 2021-06-18

**Authors:** Rosa Paola Radice, Rocco Fiorentino, Maria De Luca, Antonina Rita Limongi, Emanuele Viviano, Giovanna Bermano, Giuseppe Martelli

**Affiliations:** a University of Basilicata, Viale dell'AteneoLucano, 1 85100 Potenza (Pz), Italy; b Bioinnova s.r.l.s, via Ponte Nove Luci 9, 85100 Potenza (Pz), Italy; c Centre for Obesity Research and Education (CORE), School of Pharmacy and Life Sciences, Robert Gordon University, Aberdeen United Kingdom; d ALMACABIO Srl, C/so Italia 27, 39100 Bolzano, Italy; e Thema Informatik s.r.l., Via Ressel 2/F, 39100 Bolzano, Italy; f Department of science, University of Basilicata, via dell'ateneo lucano 10

**Keywords:** Astaxanthin, Haematococcus pluvialis, Food supplements, Innovative methodology, Microalgae, Growth optimization

## Abstract

*H. pluvialis* is a green unicellular microalgae and it is the first producer of natural astaxanthin in the world if subjected to stress conditions such as high light, high salinity and nutrient starvation. Astaxanthin is a powerful antioxidant used in many fields, such as aquaculture, pharmaceutical, food supplements and cosmetic. To obtain a large amount of astaxanthin, researcher focused on the optimisation of *H. pluvialis* growth. *H. pluvialis* has four different size growth stage (macrozooids, microzooids, palmelloid and “red non-motile astaxanthin accumulated encysted”), and astaxanthin production occur in the last phase. Recent studies shown that non-motile cells can produce more astaxanthin than motile cells if subjected to light stress. For these reasons, the aim of this study is to find a new and innovative methodology to select and recovery *H. pluvialis* in his last growth phase thanks to an electrophoretic run, and optimize, in this way, astaxanthin production.

## Highlights

• H. pluvialis non-motile cells produce more astaxanthin.
• H. pluvialis cells could be separated, based on their size, by an electric field.
• H. pluvialis non-motile cells are bigger than motile cells, and it's possible to recovery non-motile cells using this innovative protocol.

## 1 Introduction

Today, scientific interest has focused on the use of microalgae as bioreactors of useful substances. There are many species of microalgae that are used in both the pharmaceutical and food sectors. The main challenges for the industrial use of microalgae relate to their cultivation. For this reason, many studies have been carried out to understand how to balance the different nutrients required by cells to optimize the production of the desired metabolites and how to setup physical parameters to guarantee high biomass accumulation [1], [2], [3], [4]. *Haematococcus pluvialis* is a green unicellular microalgae belonging to *Chlorophyceae* class that grows in different world area [5]. Over the last year, effort have been made to study growth conditions and ability to produce astaxanthin, to find optimum growth conditions to obtain high cell productivity and to recover high amount of biomass [6]. *H. pluvialis* is the best producer of astaxanthin (3,3′- dihydroxy-ß-carotene-4,4′-dione), a red secondary carotenoid belonging to the xanthophyll family, and considered a very powerful antioxidant [7, 8]. Astaxanthin is used in different sectors including aquaculture, pharmaceutical, food supplement [9] and cosmetic [10], [11], [12], [13], and is responsible for the red/pink colour of many fish and crustaceans as *H. pluvialis* is the first step of their food chain [14]. Astaxanthin is used as an anti-ageing factor in different cosmetic formulations thanks to its anti-inflammatory properties, and its effects on skin to fight DNA damage induced by UV radiations [15], but recent studies have shown that the antioxidant action of astaxanthin is also of significant importance in the treatment of various diseases such as obesity [16], [17], [18], the treatment of neurodegenerative diseases [19], such as Parkinson's [20] and modulation of the immune system [21, 22]. *H. pluvialis* contains about 5% of astaxanthin (dry weight) and its production is regulated by different stress conditions and its related with *H, pluvialis* cell cycle [23]. Several studies have shown that high light intensity, high salinity of the medium, pH, temperature, and lack of nutrients, stimulate *H. pluvialis* to produce astaxanthin [24], [25], [26], reaching even about 7% in soils poor in phosphorus [27] or thank to a photoautotrophic induction [28]. Astaxanthin production usually occurs in two different stages [29]. A first green phase useful for the accumulation of biomass and a red phase useful for the production of astaxanthin [30]. *H. pluvialis* asexual reproduction [31] could be divided into four different stages [32]. The process starts with gametogenesis where green oval gametes with two flagella [33] called macrozooids (8–20 μm long) divide by mitosis in 2–32 cells daughters called microzooids. After the germination, small and bi-flagellated *H. pluvialis* cells settle and become palmelloid cells [7] called also coccoid cells or encystment [32, 33]. In the last life cycle phase, after the maturation, cells are called “red non-motile astaxanthin accumulated encysted” (20 - 50 μm) [7]. Cell-wall changes its composition and thickness during all the *H. pluvialis* life. Macrozooids have a very thin cell wall and the extracellular environment is characterized by a gelatinous matrix. In the successive stages, cells develop a rigid cell-wall that surround and protect the cellular body [34]. It is possible to highlight differences in cytoplasmic components in addition to the morphological differences during the *H. pluvialis* life cycle; in the vegetative stage, *H. pluvialis* contains more chlorophyll and protein then carotenoid which increases during the cyst formation [32]. Astaxanthin is accumulated in the cell centre into not visible small lipid droplet, but only under stress conditions, these droplet migrates near the cell surface to protect the cell from the stress inducted [35]. High levels of astaxanthin were detected in the cystic phase compared to the previous phases of growth even under normal non-stressful conditions [31]. Furthermore, in their study, Li et al., 2019 [36] show how astaxanthin production is higher in non-motile cells than motile cells. Their results show that the astaxanthin content in macrozooids is 1.94 times lower than non-motile cells cultures. Studies in the literature show that no methodology exploits the electrophoretic gel run to separate cells. For this reason, this study aims to develop a new technology able to separate *H. pluvialis* cells at different life cycle stages to recover non-motile cells and optimize astaxanthin production. To this extent, electrophoresis gel has been used for separation exploiting the net negative charge present on the cell wall and cellular dimensions. In this way, it's possible to optimize *H. pluvialis* growth thanks to an economical, rapid and easy methodology that permits to use of macrozooids as inoculum for biomass improvement and cysts as direct inoculum to astaxanthin production using a low stress condition.

## 2 Materials and methods

### 2.1 Algal strain and cell growth

**Fig. 1:**
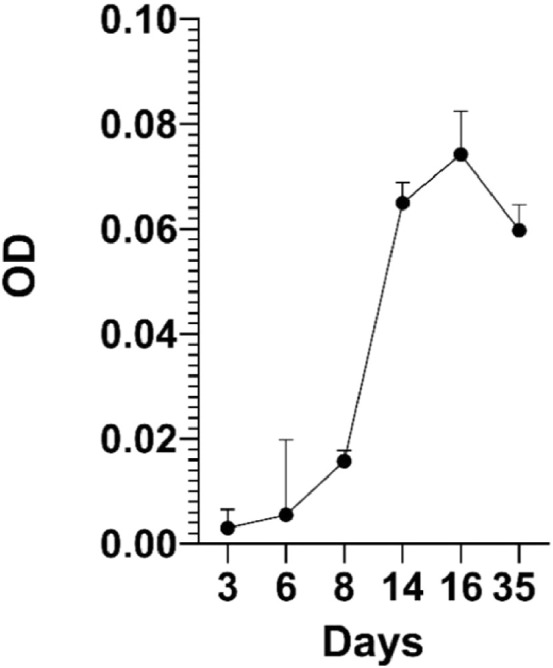
*H. pluvialis* growth curve. OD = 750 nm.

*H. pluvialis* UTEX 2505 were grown in a self-produced media produced dissolving 0.3 gr of Greenhouse Special 20–20–20 (BIOGARD) powder in 1 litre of distilled water (Greenhouse special powder contains HNO₃ 6% (w/v), NH₄⁺ 5.2% (w/v), CH_4_N_2_O 8.8% (w/v), P_2_O_5_ 20% (w/v), K_2_O 20% (w/v), B 0.05% (w/v), Cu 0.01% (w/v), Fe 0.2% (w/v), Mn 0.1%(w/v), Mo 0.005% (w/v), Zn 0.01%(w/v), chelating agent EDTA) under a light intensity of 120 mmol photons m^–2^s^–1^ on a 16 h: 8 h light/dark cycle at 25 °C. Cultures were not supplied with extra source of CO2 and were shaken by mechanical agitator (g24 environmental incubator shaker, American Laboratory Trading) at 70 rpm. Algal growth was assessed by measuring optical density at 750 nm (Fig. 1) (SPECTROstar® Nano, BMG Labtech) and cell counts by light microscopy (Zeiss Axioplan) using the Burker chamber (BLAUBRAND).

### 2.2 Gel electrophoresis

To obtain a good and solid enough support for cells’ separation, different concentrations of agarose (1.0% - 1.5% - 2.0%) were tested for gel preparation. Agarose gel (EMR010001, EuroClone S.p.a., lot n. 468,654) was made up in 100 ml of growth medium (Greenhouse special), which was also used as running buffer. Specific furrows about 3 cm long, were self-produced to allow cell loading and migration. Fifty μl of cell culture, were added in the middle of the furrow and different voltages were tested to find the right condition for the run. The gel was run using PowerPac 3000, Biorad. and the whole process was checked through a stereomicroscope.

### 2.3 pH measurement

Using a digital pH-metre (pH50 + DHS bench-top pH metre, Giorgio Bormac S.r.l), pH values of the running buffer at the opposite poles of the gel were measured before and after the running.

### 2.4 Sample collection

**Fig. 2:**
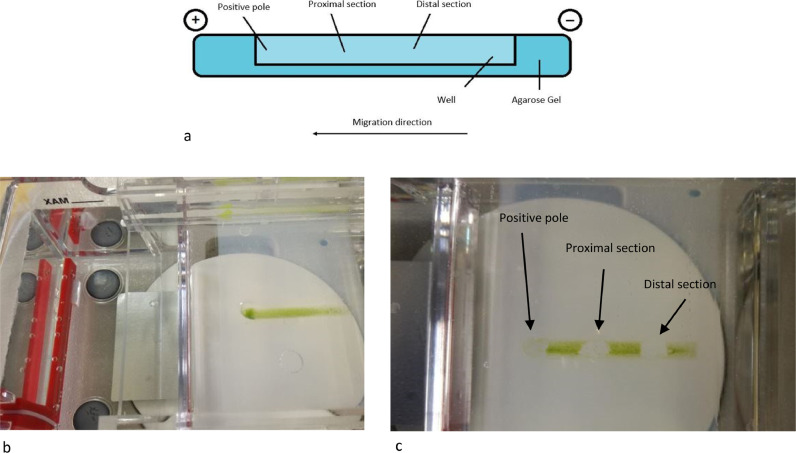
a) Schematic description of sample collection after the run. b) Agarose gel running, and cells separation. c) collection of each section: positive pole (first on the left), proximal section (centre), and distal section (first on the right).

After the run, cells were collected from three different points of the gel (positive pole, proximal section and distal section) (Fig. 2). Each collected samples were stored in the fridge and a slide was prepared which was observed under an optical microscope (Zeiss Axioplan). A subculture was obtained inoculating in 25 ml flask each collected fraction called C1, C2 and C3 for positive pole, proximal and distal section respectively. Cell density was calculated through cell count and subculture was made using 4.5 × 10^5^ cells mL^−1^.

### 2.5 Stress induction, chlorophyll and carotenoids extraction and analysis

Subcultures were grown for seven days in 25 ml of M1B5 medium following Tocquin et al. instruction [37] under low light condition to avoid cell differentiation. After seven days, subcultures were subjected to saline stress, to stimulate astaxanthin production adding 1% of NaCl. Chlorophylls (Chl α and Chl b) and carotenoids were determined through a photometrical assay. Following Boussiba and Vonshak, 1991 protocol [26], 1 ml of culture was centrifuged for 5 min at 18,000 g. The pellet was re-suspended into 1 ml of DMSO. The mixture was heated for 10 min at 70 °C. Extraction was repeated until a colourless pellet was obtained. On the supernatant, the optical density was determined at 649 nm/665 nm/480 nm. The amount of carotenoid (μg ml^−1^), including astaxanthin, was calculated according to Wellburn, 1994 [38].

Chl α = 12.19 A_665_ – 3.45 A_649_
Chl *b* = 21.99 A_649_ – 5.32 A_665_
Carotenoids = (1000 A_480_ – 2.14 Chlα – 70.16 Chl b)/220

### 2.6 Cell size assessment

Through the images acquired under the microscope (Zeiss Axioplan) and with the help of an image processing software (Fiji is just-IMAGEJ, V1.52i), analyses were carried out to size the cell populations collected following the separation.

### 2.7 Statistical analysis

The results were analysed using Microsoft Excel 2016 (Microsoft, Redmond, USA) and Graph-Pad Prism 8.0.2 (San Diego, USA). For each experiment, 180 cells were count (60 for each collection: positive pole, proximal section, distal section) and the experiments were performed 3 times for all the analyses (Fig. 5). Descriptive statistic was performed to calculate mean, standard deviation (SD) and standard error of mean (SEM) and a variance analysis was performed to estimate the statistical difference between the sample groups (One way-ANOVA test). A variance analysis was performed to compare the difference between each experiment (Two way-ANOVA). A p-value <0.05 was considered significant.

## 3 Results and dicussion

**Fig. 3:**
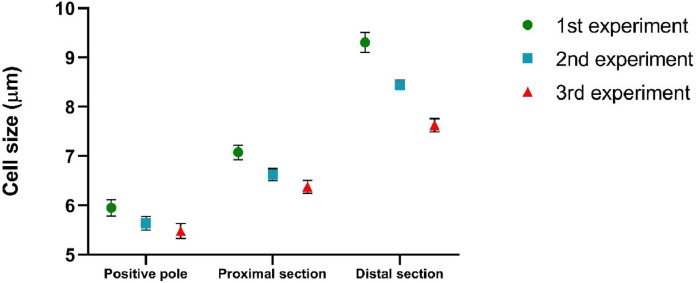
Cell size in a different section. Results shown cell size mean with SEM (p-value < 0.05). For each experiment, 180 cells were count (60 for each section).

**Fig. 4:**
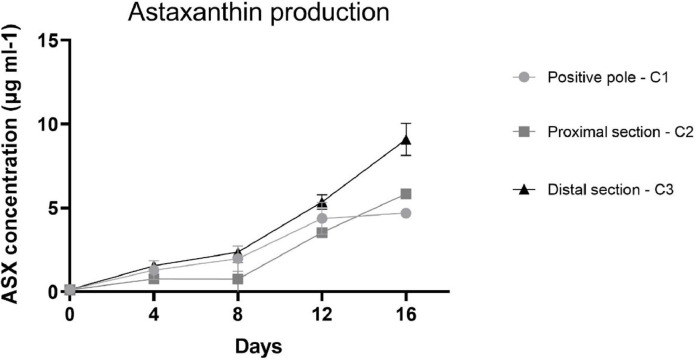
Concentration of astaxanthin produced in cultures after saline stress induction at 7th day of gel electrophoretic recovery*.*

To obtain a more heterogeneous population, samples used for electrophoretic analysis were collected from the exponential phase (Day 11 of algal growth described in Fig. 1). In this way we wanted to ensure the presence of the different growth phases, therefore both macrozooids and encysts. Different agarose concentrations were tested to find the optimal conditions to provide support to cells and for cell migration: a 2% agarose gel gave the best result. As the running buffer was mainly made of water, lower agarose concentrations were not so compact to allow cell migration. Fifty μl of cell culture, with a density equal to 1.5 × 10^7^ cells mL^−1^ were pipetted in the middle of the furrow, and cells remained in suspension without entering the gel. Only, once the electric field was applied, cells fell to the bottom of the furrow. An important parameter to affect cell migration is pH, which influence the process. The buffer solution pH was measured before subjecting the cells to electrophoresis. The solution pH was 6.8. The first tests were carried out at a voltage of 100 V. In these conditions, cells loaded in the centre of the furrow did not migrate towards either of the two poles; in fact, the pH, after a 20 min run, was 6.7 at the positive pole and 7.12 at the negative pole. Other tests were carried out applying a voltage of 200 V for 20 min. After the run, the pH was 3.28 at the positive pole and 9.54 at the negative pole. Under these conditions, cells migrated towards the positive pole, as expected: water hydrolysis produces the right amount of positive charges able to attract negative charges present on the cell wall. To our knowledge, this is the first study in which cell separation was performed using an electrophoretic gel. The innovation of the technique confirms the current knowledge on the conformation of the cell wall, in fact it is known that during the encyst phase, the cell wall is rigid and thick (1.8–2.2 μm) and it consists of different layers, including trilaminar sheath (TLS), secondary wall (SW) and tertiary wall (TW) [39]. TLS is formed by algaenan [40], an aliphatic, insoluble, resistant biopolymeric compounds [41], which could be responsible for the cell migration, having negative groups in its structure. Furthermore, the electric field separates the cell based on cell diameter at the different growth phases. After the run, cells separated and created a smear along the bottom of the furrow. Cells were collected as described in Fig. 2, with the help of a 200 μl pipette at three different point (positive pole, proximal section and distal section). Smaller cells are affected by the electric field more compared to bigger ones and, therefore, migrate faster and further. Our measurement results showed that cells size recovered near the positive pole were 2.8 times smaller than cells recovered in the distal section confirming the success of our methodology as shown in Fig. 3. Considering all the experiments conducted, cells at the positive pole have the average of 5.7 μm. Proximal cells have the average of 6.7 μm, whereas distal cells have the average of 8.5 μm. Each section collected was observed under optical microscopy and counted to have the same density for the next subculture. Cell density was adjusted to reach 4.5 × 10^5^ cells mL^−1^ and it was inoculated into 25 ml of specific growth medium to inhibit cell differentiation. A specific medium and a low light intensity was used to slows the differentiation of macrozooids cells as suggested by Tocquin et al. (2012) [37, 42]. We decided to keep the cells in a state of quiescence for seven days to evaluate whether the electric field had created some modification. It is known that the use of electrical impulses favours the modification of the metabolic pathway of astaxanthin biosynthesis. [43]. For this reason, given that the main purpose of this preliminary study is to evaluate whether the selection of *H. pluvialis* cysts is advantageous in the production of astaxanthin, the state of quiescence has allowed us to normalize the effects induced by the electric field on the production of secondary metabolites [44]. After a week cell cultures were stressed with NaCl (1%). Chlorophyll and carotenoid content was measured every 4 days over the following 16 days using dimethyl sulfoxide (DMSO) as extraction solvent. Tri-laminar sheath blocks organic solvent such as methanol or acetone but DMSO is able to penetrate cell wall and it is usually used for astaxanthin extraction [45, 46]. As reported in Fig. 4 Astaxanthin content at the start of salinity stress induction is very low in each section. After 8 days of stress, the differences between each section were not so high, but at the end, after 16 days, Astaxanthin content was equal to 4.700 μg/ml^−1^ in C1, 5.843 μg/ml^−1^ in C2 and 9.081 μg/ml^−1^ in C3. This show that astaxanthin production is 1.93 times higher in C3 culture than C1 culture.

**Fig. 5:**
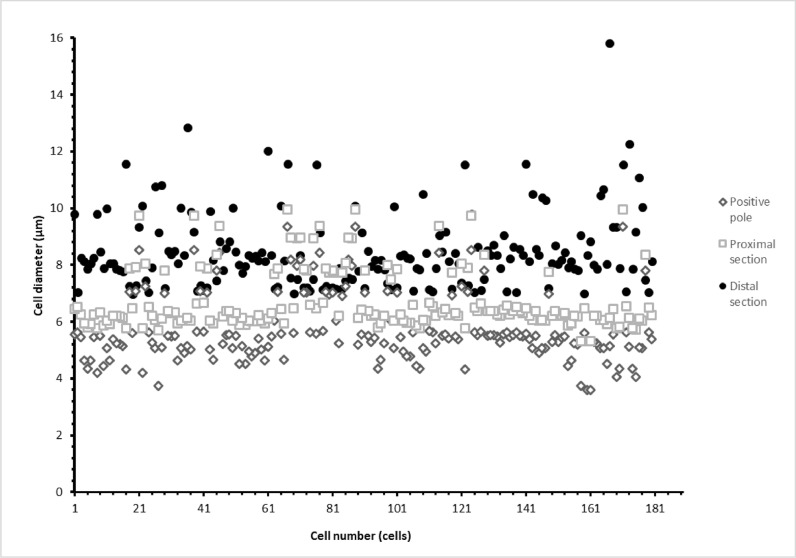
The distribution of all *H. pluvialis* cells in different diameter ranges, recovered in the three experiments in the three distinct points of the furrow.

## 4 Conclusions

*H. pluvialis* is a great producer of astaxanthin, on the strongest anti-oxidant molecule. Astaxanthin could be used for many purposes as the creation of functional foods. Recent studies show that *H. pluvialis* produces higher amounts of astaxanthin when it is in the last phase of growth. For this reason, we have tried to find a new methodology to separate the cells exploiting their diameter and the negative charges present on their wall. Our results show that submitting cells to high voltage they migrate to positive pole and separate according to cell size. In this way, it is possible to recover the higher cells (cystic phase), use them as direct inoculum for new cultures and increase astaxanthin production after stress induction. Our results were obtained starting from a fairly high cell concentration and this suggests the possibility of increasing the number of cells to be separated using even larger wells. Furthermore, the use of an electric field places the cells already in an initial state of stress which could help improve the production of astaxanthin. Our work, thus, provides the opportunity of optimizing existing *H. pluvialis*’s cultivation strategy. However, future studies should evaluate and quantify the possible modifications of the astaxanthin metabolic pathway that occurred following the application of the electric field. In this way, the methodology developed by us would be even more valid as it would allow us to separate and at the same time stress the cells, to produce a higher quantity of astaxanthin than the classical methods.

## Declaration of Competing Interest

The authors declare that they have no known competing financial interests or personal relationships that could have appeared to influence the work reported in this paper.
